# Bioactive Hydrogel Supplemented with Stromal Cell-Derived Extracellular Vesicles Enhance Wound Healing

**DOI:** 10.3390/pharmaceutics17020162

**Published:** 2025-01-25

**Authors:** Matteo Galbiati, Fabio Maiullari, Maria Grazia Ceraolo, Salma Bousselmi, Nicole Fratini, Klajdi Gega, Sandro Recchia, Anna Maria Ferretti, Giovanni Scala, Marco Costantini, Tommaso Sciarra, Roberto Rizzi, Claudia Bearzi

**Affiliations:** 1 Institute for Biomedical Technologies, National Research Council, Via Fratelli Cervi, 93, Segrate, 20054 Milan, Italy; galbiati@ingm.org (M.G.); fabio.maiullari3d@gmail.com (F.M.); claudiogega@live.com (K.G.); 2 Institute of Physical Chemistry, Polish Academy of Sciences, Marcina Kasprzaka 44/52, 01-224 Warsaw, Poland; mcostantini@ichf.edu.pl; 3 Neurology Unit, Fondazione IRCCS Ca’ Granda Ospedale Maggiore Policlinico, 20122 Milan, Italy; ceraolo@ingm.org (M.G.C.); salma.bousselmi@students.uniroma2.eu (S.B.); 4 Ph.D. Program in Cellular and Molecular Biology, Department of Biology, University of Rome “Tor Vergata”, Via della Ricerca Scientifica, 1, 00133 Rome, Italy; 5 Department of Molecular Medicine, Sapienza University, Viale Regina Elena, 324, 00161 Rome, Italy; nicole.fratini@uniroma1.it; 6 Department of Science and High Technology, University of Insubria, 22100 Como, Italy; sandro.recchia@uninsubria.it; 7 CNR-SCITEC Istituto di Scienze e Tecnologie Chimiche “G. Natta”, Via G. Fantoli 16/15, 20138 Milan, Italy; anna.ferretti@scitec.cnr.it; 8 Department of Biology, University Federico II, 80128 Naples, Italy; giovanni.scala@unina.it; 9 Joint Veteran Center, Scientific Department, Army Medical Center, 00184 Rome, Italy; tommaso.sciarra@esercito.difesa.it; 10 Department of Medical-Surgical Sciences and Biotechnologies, Sapienza University, Corso della Repubblica 79, 04100 Latina, Italy

**Keywords:** regenerative medicine, wound healing, extracellular vesicles, bioactive hydrogel, lyoprotectants

## Abstract

**Background/Objectives**: Skin regeneration is a rapidly advancing field with significant implications for regenerative medicine, particularly in treating wounds and burns. This study explores the potential of hydrogels functionalized with fibroblast-derived extracellular vesicles (EVs) to enhance skin regeneration in vivo. Being immunoprivileged, EVs minimize immune rejection, offering an attractive alternative to whole-cell therapies by replicating fibroblasts’ key roles in tissue repair. **Methods**: To promote EVs’ versatility and effective application across different conditions, a lyophilization method with lyoprotectants was optimized. Then, EVs were used to functionalize a hydrogel to perform experiments on murine cutaneous wound models. **Results**: Gelatin methacrylate (GelMA) was selected as the polymeric hydrogel due to its biocompatibility, tunable mechanical properties, and ability to support wound healing. Mechanical tests confirmed GelMA’s strength and elasticity for this application. Fibroblast-derived EVs were characterized using Western blot, Transmission Electron Microscopy, and NanoSight analysis, proving their integrity, size distribution, and stability. miRNome profiling identified enriched biological pathways related to cell migration, differentiation, and angiogenesis, emphasizing the critical role of EV cargo in promoting wound repair. In a murine model, hydrogels loaded with fibroblast-derived EVs significantly accelerated wound healing compared to controls (mean wound area 0.62 mm^2^ and 4.4 mm^2^, respectively), with faster closure, enhanced epithelialization, increased vascularization, and reduced fibrosis. Notably, the lyoprotectants successfully preserved the EVs’ structure and bioactivity during freeze-drying, reducing EVs loss by 35% compared to the control group and underscoring the feasibility of this approach for long-term storage and clinical application. **Conclusions**: This study introduces a novel scalable and adaptable strategy for regenerative medicine by combining fibroblast-derived EVs with GelMA, optimizing EVs’ stability and functionality for enhanced wound healing in clinical settings, even in challenging contexts such as combat zones or large-scale natural disasters.

## 1. Introduction

Regenerative medicine and tissue engineering have transformed healthcare by introducing advanced solutions for tissue repair and organ regeneration [1,2,3]. Wound healing is a pivotal area of research, given its significant influence on patient recovery and healthcare expenses. Despite significant advancements, improving wound healing remains challenging, particularly in managing scar formation and reducing infection risks [4]. The wound healing process is complex, involving coordinated cellular activities such as migration, proliferation, extracellular matrix remodeling, angiogenesis, and re-epithelialization [5]. However, natural healing is often slow, and various factors, including chronic inflammation, infections, and pre-existing medical conditions, can hinder effective wound closure, leading to scarring that may progress to pathological fibrosis. Several traditional approaches, including laser therapies [6,7], growth factor-based treatments [8], and cell-based therapies [9,10,11], have been explored to enhance wound healing. While promising, these methods face notable limitations, such as invasiveness, low effectiveness, risks of tumorigenicity, and potential immune rejection, which limit their clinical utility.

In recent years, advances in bioengineering have driven the development of biomaterials specifically designed to support regeneration [12]. Among these, hydrogels have emerged as one of the most promising due to their biocompatibility, antibacterial properties, and ease of application [13,14]. These three-dimensional scaffolds absorb large amounts of water, creating an ideal environment for cellular proliferation and enabling the controlled delivery of therapeutic agents directly to the wound site [15,16]. Although hydrogels have typically been studied when combined with cells, recent advancements are shifting toward functionalizing these biomaterials with bioactive molecules to enhance their efficacy [17,18]. An in vivo study revealed that hydrogel scaffolds functionalized with Cannabidiol-Loaded Lipid Nanoparticles improved dermal architecture, collagen deposition, enhanced angiogenesis, and reduced inflammation, thus significantly accelerating wound healing [19].

Our study explores an innovative strategy to enhance wound healing by functionalizing hydrogels with extracellular vesicles (EVs), which we hypothesize could yield improved therapeutic outcomes. EVs are nanosized particles secreted by various cell types that mediate intercellular communication and have demonstrated remarkable potential in tissue regeneration [20,21,22]. These vesicles function as key mediators in numerous essential biological processes, carrying a rich and heterogeneous cargo that includes growth factors, proteins, cytokines, lipids, and nucleic acids. EVs have emerged as a promising cell-free alternative for therapeutic applications. Unlike whole-cell therapies, EVs have several advantages, including reduced immunogenicity, making them an immunoprivileged approach. Furthermore, EVs retain the bioactive properties of their originating cells without the risks associated with direct cell transplantation [23]. Exploiting the complexity of their cargo, which to date is almost impossible to replicate in vitro, can open important scenarios and overcome the limitations of using single factors as bioactive molecules. In a previous study, we demonstrated the therapeutic potential of EVs derived from HUVECs to enhance tissue regeneration following myocardial infarction [24], laying the groundwork for exploring this approach in other pathological contexts.

One of the primary challenges in translating EVs-based therapies to the clinic is the preservation and storage of these particles without compromising their biological activity. Lyophilization has shown promise as a technique for maintaining the structural integrity and bioactivity of EVs during long-term storage at ambient temperatures [25]. By stabilizing EVs through lyophilization, their potential use in diverse clinical settings, including resource-limited environments, becomes more feasible [26]. Despite the limited studies on EV lyophilization, this strategy holds great promise for preserving their stability and functionality, making them more suitable for storage and therapeutic use. However, lyophilization can pose challenges, such as EV degradation or a loss of functionality due to stresses like ice crystallization and protein denaturation. To overcome these hurdles, lyoprotectants, which are substances that shield biological materials during freeze-drying, have proven to be indispensable [27,28]. These protective agents stabilize EVs membranes and prevent protein aggregation, ensuring the preservation of their structural and functional integrity throughout the process [29]. Motivated by these advantages, we sought to evaluate the effectiveness of lyophilization for EVs preservation, aiming to establish a reliable method to maintain their bioactivity and potential for clinical applications.

In this study, Gelatin methacrylate (GelMA) was selected to deliver and support the EVs in skin wound healing. GelMA hydrogels mimic the natural extracellular matrix, offering structural support for cell growth and tissue regeneration [30]; they are particularly suitable due to their adjustable mechanical properties, biocompatibility, and biodegradability, making them ideal for wound healing. Additionally, they act as a protective matrix for EVs, allowing sustained release and reducing rapid degradation in biological fluids [18]. This controlled release helps to maintain the bioactivity of EVs components, enhancing their therapeutic potential in tissue repair. Beyond structural support, these biomaterials also provide cell-anchoring sites that facilitate cell migration and proliferation, essential for effective tissue regeneration [31,32].

Primary human dermal fibroblasts were used as the source of the EVs, as they play a central role in wound healing. These cells secrete essential extracellular matrix components, growth factors, and cytokines that are critical for tissue repair [33,34]. They are pivotal in synthesizing and remodeling the matrix by producing collagen, elastin, and glycoproteins alongside signaling molecules that drive regeneration [35,36]. Additionally, fibroblasts are readily isolable and highly adaptable to culture conditions, making them accessible for research and therapeutic use and enabling innovative wound healing strategies. Thus, fibroblast-derived EVs could potentially replicate many essential functions of fibroblasts by encapsulating bioactive molecules that mimic their paracrine signaling. This approach may enhance angiogenesis, cell proliferation, and matrix remodeling, positioning EVs as promising therapeutic agents that offer the advantages of fibroblasts without the limitations of whole-cell therapies, such as storage challenges and immune rejection [37].

Based on these premises, the present work aims to address EVs preservation and delivery challenges by exploring lyophilized fibroblast-derived EVs within GelMA hydrogels for improved wound healing. We expect this combination to synergistically boost tissue regeneration, accelerate wound closure, and improve tissue properties and functionality.

## 2. Materials and Methods

### 2.1. Human Fibroblasts Culture

Primary human dermal fibroblasts were grown in a complete growth medium, composed of Dulbecco’s Modified Eagle Medium High Glucose (DMEM, L0101-500, BioWest, Nuaillé, France) supplemented with 10% Fetal Bovine Serum (FBS, 10270106, Gibco, Waltham, MA, USA), 1% penicillin/streptomycin (P/S stock solution 10,000 U/mL, 15140122, Gibco), and 1% Sodium Pyruvate (100 mM, 11360070, Gibco) at 37 °C and 5% CO_2_. When the cells achieved 70–80% confluence, they were collected through trypsinization and subsequently expanded.

### 2.2. Isolation of Human Fibroblast-Derived EVs

Human dermal fibroblasts (ATCC) were seeded in 150 mm dishes and cultured in the complete growth medium. Once the cells reached a confluence state of ∼60%, they were washed twice with sterile phosphate buffer saline (PBS, L0615-500, BioWest) and then cultured in an EV-free complete medium for 48 h. FBS was ultracentrifuged to remove EVs and obtain EV-free FBS, which was then used to prepare the EV-free complete medium. The EVs were pelleted using a Beckman L-90 centrifuge (Beckman Instruments, Inc., Fullerton, CA, USA) equipped with a Ti-70 rotor (Beckman Instruments) at 125,000× *g* for 90 min at 4 °C using 25 mL polycarbonate tubes (Beckman Coulter, Brea, CA, USA). After 48 h of culture, the conditioned medium of fibroblasts was collected from each dish and centrifuged at 500× *g* for 15 min and subsequently at 2000× *g* for 25 min at 4 °C to remove cell debris and organelles. Afterwards, the supernatants were transferred into 25 mL polycarbonate tubes and ultracentrifugated at 125,000× *g* for 90 min at 4 °C. Next, the supernatants were discarded and the obtained EVs pellets were resuspended in 1.25 mL of sterile PBS and collected in 1.5 mL polycarbonate. To concentrate the EVs into a smaller volume, another ultracentrifuge was performed at 125,000× *g* for 90 min at 4 °C. The pellets were resuspended either in 80 µL of PBS for NanoSight and TEM analysis, 80 µL of RIPA buffer for protein extraction for Western blot analysis, or in 700 µL of Qiazol for miRNome analysis.

### 2.3. Western Blotting

Western blotting was performed using a sodium dodecyl sulfate–polyacrylamide gel electrophoresis (SDS–PAGE) system. EVs were lysed in RIPA buffer, containing 50 mM Tris HCl pH 7.6, 150 mM NaCl, 0.5% sodium deoxycholate (264103, Merck Millipore, Burlington, MA, USA), 0.1% sodium dodecyl sulfate (SDS, 10% #1610416, BIO RAD), 1% Triton X-100 (Sigma-Aldrich), 1 mM EDTA (0.5 M, 324506, Merck Millipore), protease inhibitor cocktail (04693116001, Roche, Basel, Switzerland), and 1 mM phenylmethylsulphonyl fluoride (36978, Thermo Scientific, Waltham, MA, USA). Proteins were quantified with Qubit Protein Assay Kits (Q33212, Invitrogen, Carlsbad, CA, USA), and 20 µg were loaded on Bolt 4–12% Bis-Tris gel (NW04125BOX, Thermo Fisher Scientific, Waltham, MA, USA), transferred to nitrocellulose membranes (GE10600079, Amersham Protran, Johns Creek, GA, USA), and blocked with 5% non-fat dry milk powder (1.15363, Merck Millipore) in 1X Tris-buffered saline. Membranes were incubated with the following primary antibodies: anti-CD9 (1:1000, Cell Signaling, Danvers, MA, USA), anti-CD81 (1:100, Cell Signaling), anti-TSG101 (1:1000, Abcam, Cambridge, UK), and then with the corresponding anti-rabbit peroxidase-linked secondary antibodies (1:2000, GE Healthcare Life Sciences, Havelock, NE, USA). Signal detection was performed via chemiluminescence reaction (ECL, GE Healthcare) using the iBright (Thermo Fischer Scientific) Western blot imaging system.

### 2.4. NanoSight Analysis

Nanoparticle Tracking Analysis was performed using a NanoSight NS300 instrument (Malvern Panalytical, Malvern, UK). Five 30 s videos were recorded for each sample with a camera level set at 14/15 and a detection threshold set at 2. The EVs concentration and size distribution were subsequently analyzed with NTA 3.2 software.

### 2.5. Transmission Electron Microscope Acquisition

Vesicles isolated from the three experimental conditions were analyzed with a Zeiss LIBRA 200FE-HR TEM, operating at 200 kV and equipped with a second-generation in-column W filter. Sample preparation followed the freeze-drying protocol previously described. A drop of vesicle suspension (3 × 10^6^ particles/µL) was placed onto a Carbon/Formvar TEM grid, left to air-dry, and then stained with UranyLess (EMS-Electron Microscopy Science, St. Louis, MO, USA).

### 2.6. EVs Freeze-Drying

Trehalose (182550250, Thermo Fisher Scientific) and PVP40 (PVP40-50G, Sigma Aldrich, St. Louis, MO, USA) were used as lyoprotectants in the preparation of the freeze-drying solution. To maintain the overall osmolality of the lyophilization solutions at around 290 mOsm/kg, the strength of PBS was adjusted according to trehalose concentration, while PVP40 concentration was excluded due to its irrelevant contribution to the overall osmolality. The final concentrations of trehalose and PVP40 were 100 mM and 5%, respectively. The EV samples were divided into two groups and resuspended in either 600 µL of lyophilization solution with lyoprotectants or in 600 µL µl of PBS. The EV samples were placed in safe-lock polypropylene microcentrifuge tubes and were frozen overnight at −80 °C. The samples were closed with a perforated parafilm membrane immediately before being loaded into the Benchtop Freeze Dryer (LIO-5P, 5pascal, Trezzano sul Naviglio, Milan, Italy). The samples were lyophilized overnight and stored at −80 °C. Before use, the lyophilized EVs were rehydrated with bidistilled water to restore the original volume and then resuspended. Next, trehalose and PVP40 were removed by centrifugation with concentrator tubes (UFC905024, Merck, St. Louis, MO, USA) with a 50 kDa MWCO membrane, and NanoSight analysis was performed.

### 2.7. miRNome Analysis

Total RNA was extracted from fibroblast-derived EVs (n = 3) using the commercial miRNeasy Mini Kit (217004, Qiagen, Hilden, Germania). The concentration and purity of the total RNA were assessed using Nanodrop 2000 and Bioanalyzer 2100. Samples were then sequenced using NGS technology. Briefly, 3′ and 5′ adaptors were ligated to 3′ and 5′ end of small RNA, respectively. Then, the first strand cDNA was synthesized after hybridization with reverse transcription primer. The double-stranded cDNA library was generated through PCR enrichment. After purification and size selection, libraries with insertions between 18~40 bp were ready for sequencing with Illumina Sequencing SE50, generating approximately 20 million reads. The NGS data were generated by Novogene Co., Ltd. (Cambridge, UK) Small RNA sequencing data for the three replicates were processed using the nf-core/smrnaseq pipeline version 2.4.0 [38]. In particular, FastP [39] was used to perform adapter trimming and quality and length filtering on raw fastq files. Bowtie1 [40] was used to perform the alignment of filtered reads against miRBase mature miRNA catalog. Samtools [41] was used for the sorting and indexing of BAM files and to quantify miRBase miRNA abundances. Edger was used to compute normalized counts [42]. RNA categories for each sample were obtained using the miRTace tool [43]. Gene Ontology enrichment analysis was performed using the miEAA [44] online tool. In particular, the miEAA “miRNA enrichment analysis ((G)SEA)” was performed by passing an ordered miRNA id list based on their average TPM values among the three replicates and choosing the following parameters: annotations derived from the miRTarBase (Gene Ontology Consortium) as the category, the Bonferroni adjustment method used for multiple testing, a minimum of at least 2 hits per sub-category, and 0.05 as the significance threshold.

### 2.8. Bioactive Hydrogel Formulation

Gelatin methacrylate (GelMA) was prepared following a previously published protocol [18]. Briefly, gelatin (type A3 from porcine skin; Sigma-Aldrich, 9000-70-8) was reacted with methacrylic anhydride. Gelatin was dissolved in phosphate buffer (1 g/10 mL, pH 7.5) at 50 °C, and 0.8 mL methacrylic anhydride was added dropwise under vigorous stirring. After 2 h of reaction, the mixture was diluted, dialyzed (MWCO = 2 kDa) against distilled water for 3 days at 40 °C, and subsequently freeze-dried. The bioink stock solution was prepared by dissolving 7% of GelMA *w*/*v* in 25 mM HEPES buffer (Sigma-Aldrich, 7365-45-9). After biopolymer dissolution, the pH of the polymeric stock solution was brought to 7.2 and filtered (0.22 µm) to guarantee sterile conditions. To obtain a 5% GelMA working solution, the stock solution (GelMA 7%) was diluted with phosphate buffer or phosphate buffer + EVs at a final concentration of 9 × 10^9^ particles/mL. Thus, we obtained a control hydrogel (GelMA) and a functionalized hydrogel (GelMA + EVs), respectively. The EVs’ concentration was obtained by analyzing and quantifying through NanoSight technology, which allowed the precise measurement of the particle amount (Section 2.4 of materials and methods). Finally, photopolymerization was achieved by adding 1 mg/mL of Irgacure 2959 to the bioinks.

### 2.9. Scanning Electron Microscopy (SEM) Analysis

The SEM imaging of the polymer, both with and without EVs, was carried out using an FEI ESEM-FEG XL30 microscope. The parameters were set as described in a previous study [18]. The system was operated at an acceleration voltage of 10 kV in low vacuum mode, with the chamber pressure maintained at 1 torr and equipped with a gas secondary electron detector. Imaging conditions were optimized, including a working distance of 8 mm, a spot size of 3, and a sample temperature of 20 °C. Samples were individually mounted on stubs using carbon-based conductive double-sided adhesive tape. The samples remained stable under the electron beam without requiring cooling, even at magnifications reaching 40,000×.

### 2.10. Bioactive Hydrogel Mechanical Testing

Compressive stress–strain measurements were conducted as previously described [45]. In short, samples including 5% GelMA (*w*/*v*) and 5% GelMA (*w*/*v*) with EVs (9 × 10^9^ particles/mL) were both cross-linked under UV light at 365 nm (intensity of 4–5 mW/cm^2^) for 5 min. Compression testing was performed using a DMA Q800 Dynamic Mechanical Analyzer (TA Instruments, New Castle, DE, USA) operating in strain rate mode. The tests were conducted at a constant temperature of 25 °C under the following conditions: an initial preloading force of 0.003 N, a strain rate of 5% per minute, and a maximum deformation of 30%. Young’s modulus was calculated from the linear portion of the stress–strain curve corresponding to 0–5% strain. All experiments were conducted in triplicate, and the data are reported as mean values accompanied by standard deviation.

### 2.11. PDMS O-Ring Preparation

O-rings for in vivo implants were prepared using polydimethylsiloxane (PDMS). The PDMS was prepared by mixing the Silicone Elastomer Base (Sylgard 184) and Silicone Elastomer Curing Agent in a 10:1 ratio. The obtained mixture was poured into a 1 mm thick polycarbonate mold designed to create a PDMS sheet. After degassing to remove the air bubbles, the molds were placed in a stove at 65 °C for 3 h. Finally, the O-rings were prepared using punches with a defined diameter. For the external circumference, a punch with a diameter of 1 cm was used, while for the internal circumference, a punch with a diameter of 5 mm.

### 2.12. Cutaneous Implantation in Mice

**Figure 1:**
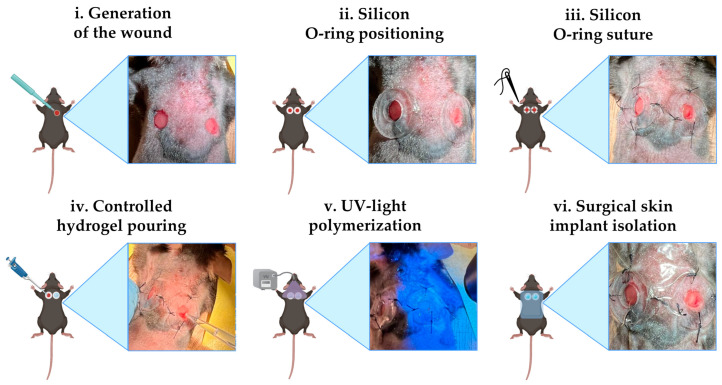
Surgical procedure. Schematic representation of the various steps of the protocol for cutaneous implantation in mice (created with Biorender.com) alongside the real image. Limited skin incisions on the back medial sides were performed with biopsy punches with a diameter of 4 mm (**i**); an O-ring with a 5 mm diameter was placed at each wound (**ii**) and then was fixed with surgical glue and four stitches (**iii**). In every wound, 20 µL of bioink was administered (**iv**). GelMA hydrogels were polymerized using a UV gun (**v**). The implants were covered and protected by a transparent water-repellent wound plaster (**vi**).

C57/BL6 mice (Jackson Laboratory, Bar Harbor, ME, USA) were used to evaluate the in vivo induced skin regeneration through EVs-functionalized hydrogel. Four-month-old male mice (n = 9) were anesthetized by 2% isoflurane inhalation. The backs of the animals were carefully shaved with an electric shaver and sterilized with ethanol solution. Afterward, the mice were placed on the operating table. Two limited skin incisions on the back medial sides were performed with biopsy punches with a diameter of 4 mm. An O-ring with a 5 mm diameter was placed on each wound, secured with surgical glue, and sutured with four stitches using 4-0 suture thread (Ethicon, Raritan, NJ, USA). The experimental conditions were the following: (i) saline solution (NT), (ii) GelMA, and (iii) GelMA + EVs. Three animals for a total of six wounds were considered for each treatment. The cross-linking of the hydrogel was performed using a UV gun (Electro-Lite Corporation mod. LED-200, Bethel, CT, USA) for 2 min. After the treatment, the implants were covered and protected by a transparent water-repellent wound plaster (Tegaderm, 3M, London, UK). The surgical procedure is reported in Figure 1. Mice were housed individually for 15 days. Finally, the mice were sacrificed via cervical dislocation, and the samples along with the surrounding tissue were excised and processed for histological and molecular analysis. All experiments involving animals were conducted according to the protocols of good animal experimentation under the Italian Health Minister approval n° 324/2024-PR-Risp. a prot. 8C65E.13-3 April 2024.

### 2.13. Confocal Microscopy Acquisition

The fibroblasts were expanded in µ-Slide (80 426, Ibidi, Gräfelfing, Germany) and when they reached 80% of confluence they were fixed in 4% Paraformaldehyde (PFA, 047347.9M1, Thermo Scientific) for 15 min at room temperature and washed three times with PBS and then stained with Vimentin (Ms, V5255, Sigma) for 2 h at room temperature; this was followed by the secondary antibody (1:1000, A32766, Invitrogen) for 1 h at room temperature. DAPI (4′, 6-Diamidino-2-Phenylindole, D1306, Life Technology, Carlsbad, CA, USA) was used for labeling the nuclei. The EVs were stained with a deep-red cell mask (C10046, Invitrogen), then ultracentrifuged and used to produce the bioactive bioink. A 50 µL sample was polymerized using UV light (VILBER LOURMAT mod. VL-6.L) at 365 nm (intensity 4–5 mW/cm^2^) for 2 min and imaged using confocal microscopy to assess the distribution of EVs within the hydrogel. The images of the samples were acquired using a Leica Confocal Microscope (Leica Microsystem Srl, Buccinasco, Milan, Italy).

### 2.14. H&E Staining and Masson’s Trichrome

The skin tissues were collected on day 14 and fixed in 4% PFA. Subsequently, the tissues were embedded in Tissue Freezing Medium Optimal Cutting Temperature (OCT, 14020108926, Leica Biosystem, Buccinasco, Milan, Italy) and sectioned using a cryostat (Leica Microsystem, Wetzlar, Germania). The sections (10-µm) were stained with Masson’s Trichrome KIT (04-010802, BioOptica, Milan, Italy) according to the manufacturer’s protocol, or using a hematoxylin and eosin KIT (BioOptica). Healthy skin (WT) sections taken from the same animals were used as controls.

### 2.15. Immunofluorescence Assay

OCT-embedded skin sections were first washed with PBS and then permeabilized with 0.2% Triton X-100 to gain access to the intracellular antigens. Sections were then incubated in 5% Bovine Serum Albumin (BSA, A1470, Sigma-Aldrich) blocking solution for 3 h to saturate the nonspecific sites, and thereafter with anti-smooth muscle actin (α-SMA, 1:1000, A2547, Sigma-Aldrich) and anti-Ki-67 (Ki-67, 1:1000, AB15580, Abcam) primary antibody diluted in 0.5% BSA solution overnight at 4 °C. Subsequently, the primary antibody solution was removed, and slides were washed two times in PBS for 5 min and incubated with 488-conjugated Isolectin B4 antibody (S-L2895, Sigma-Aldrich) and fluorescent-conjugated secondary antibodies (1:1000, A10042, Invitrogen) for 2 h. Cell nuclei were counterstained with DAPI (4′, 6-Diamidino-2-Phenylindole, D1306, Life Technology), and the slides were mounted with ProLong Glass Antifade Mountant (P36984, Invitrogen). Healthy skin (WT) sections taken from the same animals were used as controls. A TCS SP5 confocal microscope was employed to acquire the labeled sample images.

### 2.16. Statistical Analysis

Statistical analysis was carried out using Prism 5 software (GraphPad, La Jolla, CA, USA). Data are presented as mean ± SD (Standard Deviation). Differences between the sample mean at each time point were evaluated with Two-Way ANOVA. A *p*-value < 0.05 was considered statistically significant.

## 3. Results and Discussion

### 3.1. Isolation and Characterization of Human Fibroblast-Derived EVs

**Figure 2:**
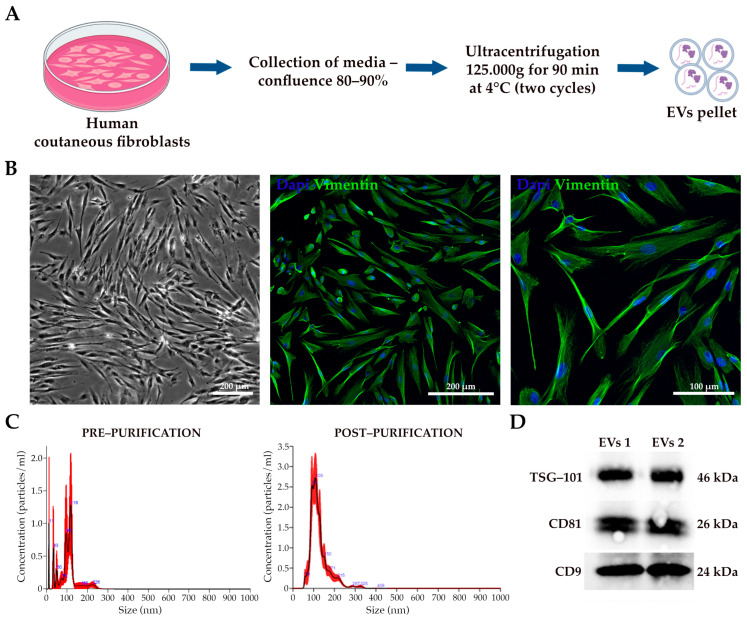
EVs characterization. (**A**) Schematic diagram of EVs isolation from fibroblasts (generated with Biorender.com). (**B**) Bright-field and immunofluorescence representative acquisition of human cutaneous fibroblasts. The scale bar represents 200 and 100 µm. (**C**) The size distribution and concentration profiles of fibroblast-derived EVs analyzed by NanoSight NS300 before and after purification. (**D**) Anti-CD9, anti-CD81, and anti-TSG-101 antibodies were used for the Western blot analysis of fibroblast-derived EVs.

EVs were successfully isolated from the conditioned medium of human dermal fibroblasts (Figure 2A). To assess the proper morphology and cell type-specific marker expression, we stained them with Vimentin, which allowed us to confirm the expected elongated and spindle-like morphology and the positivity for the fibroblast marker (Figure 2B). The pellet of EVs was re-suspended, and the vesicles were characterized using NanoSight NS300 and Western blot analysis. The purified EVs had a size range of 100 to 150 nm with a homogeneous distribution, similar to the EVs present in the supernatant prior to isolation. However, the concentration of vesicles in the supernatant was significantly lower, with a peak of 1.3 × 10^6^ particles/mL, compared to the higher peak concentration of 2.7 × 10^8^ particles/mL observed in the sample after purification (Figure 2C). Notably, the isolated population was significantly enriched in exosomes, identified by their size of 40–120 nm [46,47]. Western blot analysis confirmed the presence of tetraspanin proteins (CD9, CD81, and TSG-101), which are hallmark markers of EVs, further validating the successful isolation of these vesicles (Figure 2D). The confirmation of tetraspanin expression reflects the proper biogenesis of EVs and aligns with the previously reported data in the literature, supporting their role in cell-to-cell communication, their relevance in biological processes [48], and their suitability for downstream applications in tissue engineering and regenerative medicine fields.

### 3.2. Effects of Freeze-Drying on Fibroblast-Derived EVs

**Figure 3:**
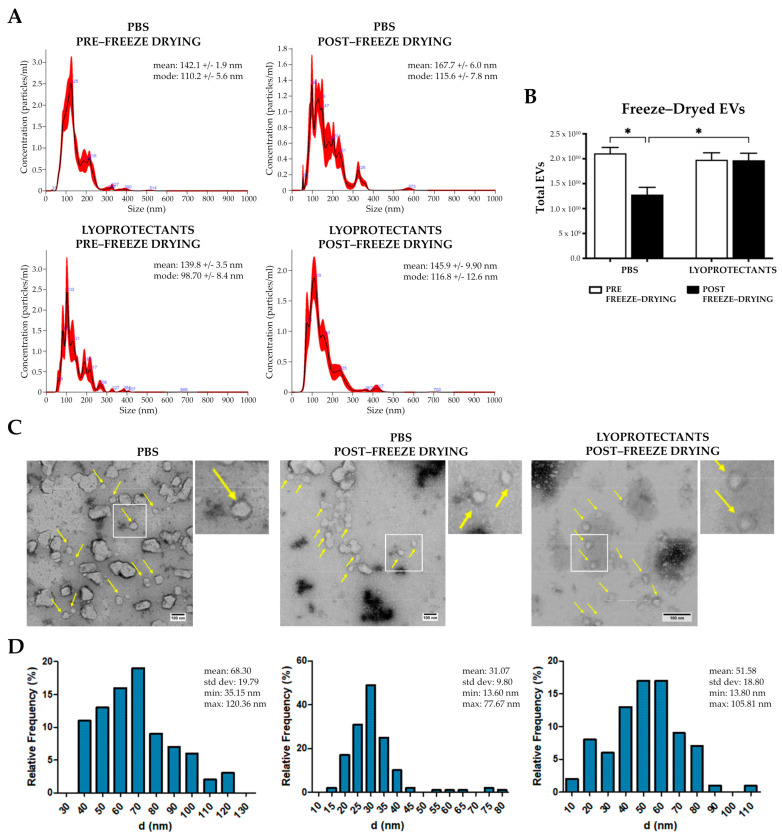
EVs freeze-drying analysis. (**A**) Size distribution profiles of fibroblast-derived EVs pre- and post-lyophilization with and without lyoprotectants analyzed by NanoSight NS300. (**B**) Histogram of EV total number in different experimental conditions, pre- and post-lyophilization. Error bars represent ± SD. Two-Way ANOVA, * *p* < 0.05. (**C**) Representative TEM images of the EVs in the three different experimental conditions. Yellow arrows show the vesicles. (**D**) Histograms displaying the diameter (nm) distribution of vesicles obtained from the diverse experimental contexts.

Freeze-drying is a critical method for the long-term preservation of EVs, yet it poses challenges to maintaining vesicle integrity. In this study, the lyoprotectants trehalose and PVP40 were used for their ability to stabilize the lipid bilayer and prevent ice crystal formation. The selection of these specific lyoprotectants and their optimal concentrations (100 mM trehalose and 5% PVP40) for the freezing-drying process were selected based on evidence from the literature [29]. Three groups of EVs were analyzed: (i) EVs resuspended in PBS and frozen, (ii) EVs resuspended in PBS and freeze-dried, and (iii) EVs resuspended with lyoprotectants (trehalose and PVP-40) and freeze-dried. Macroscopically, the lyophilized pellet in the lyoprotectant group appeared more cohesive and denser compared to the PBS group. Post-freeze-drying, the EVs were rehydrated and analyzed using NanoSight NS300 and TEM. NanoSight revealed a heterogeneous size distribution in the PBS group, with a statistically significant 35% loss of EVs after freeze-drying (Figure 3A,B), from a total particle number of approximately 2 × 10^10^ to 1 × 10^10^ (Figure 3B). In contrast, no significant changes in size or concentration were observed in the group with lyoprotectants. Indeed, the total number of pre- and post-freeze-drying EVs remained almost unchanged at roughly 2 × 10^10^ particles, indicating that adding lyoprotectants preserved the structure of the EVs. TEM analysis confirmed these results, revealing that freeze-dried EVs without lyoprotectants had a smaller diameter compared to the other groups (Figure 3C,D). A high percentage of the freeze-drying EVs without lyoprotectants were around 30 nm in size, with diameters ranging from a minimum of 13.60 nm to a maximum of 77.67 nm, probably due to vesicle fragmentation (Figure 3C,D). On the other hand, the group treated with lyoprotectants maintained a more uniform population of vesicles, with a similar diameter distribution to the control (Figure 3C,D). The peak frequency is around 55 nm, while the maximum and minimum are 105.81 nm and 13.80 nm, respectively. These results highlight the importance of lyoprotectants in maintaining vesicle integrity, as their absence leads to increased vesicle rupture and the formation of smaller particles [49]. Overall, these data demonstrate that lyoprotectants are essential for preserving EVs’ structural and functional characteristics during freeze-drying, ensuring their long-term stability.

### 3.3. miRNome Profiling of Fibroblast-Derived EVs

**Figure 4:**
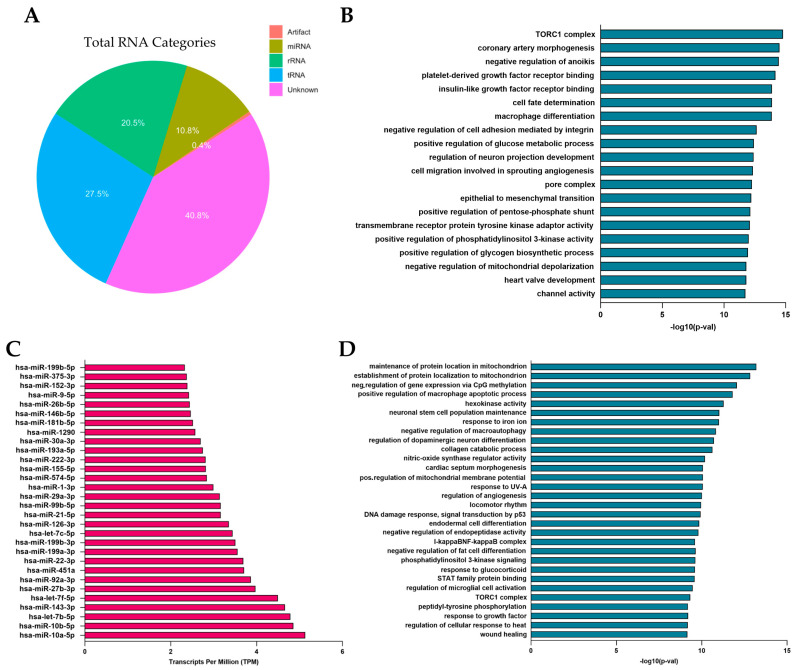
miRNome characterization of EVs derived from human dermal fibroblasts. (**A**) Pie chart describing the percentages of RNA within vesicles. (**B**) Enrichment analysis of total miRNAs using (Gene) Set Enrichment Analysis ((G)SEA). (**C**) Graphical representation of 30 selected miRNAs, identified for their key roles in the wound healing process. (**D**) Over-Representation Analysis (ORA) performed on the selected miRNAs.

We conducted an in-depth molecular characterization of fibroblast-derived EVs using miRNome profiling to evaluate their potential as bioadditives for enhancing hydrogel functionality. A comprehensive analysis of vesicle contents revealed the distribution of nucleic acids, including the proportions of miRNA (10.8%), tRNA (27.5%), and rRNA (20.5%) (Figure 4A). To identify the most abundant miRNAs, we quantified the expression levels of mature miRNAs using normalized TPM (Transcripts Per Million) values obtained from our sequencing data. The normalization process accounted for library size and sequencing depth, enabling a reliable comparison of miRNA abundance across samples. We compiled a table reporting the TPM values for all detected mature miRNAs and ranked them based on their expression levels. This allowed us to identify the top expressed miRNAs, which were considered for further pathway enrichment analyses. To explore the biological implications of these miRNAs, we performed miRNA enrichment analysis using the Gene Set Enrichment Analysis (GSEA) module of the MiEAA tool. The results revealed enriched biological pathways associated with the overall miRNA expression profile, including cell migration, differentiation, and angiogenesis (Figure 4B). Based on these findings, and through an analysis utilizing the miRBase database, we identified 30 miRNAs with critical roles in these processes (Figure 4C). Subsequent Over-Representation Analysis (ORA) further highlighted the principal pathways that involved these miRNAs (Figure 4D). We focused on four highly expressed miRNAs, including miR-10a-5p, miR-10b-5p, miR-29a-3p, and miR-21-5p, which play crucial roles in angiogenesis, extracellular matrix remodeling, and cell proliferation—processes essential for tissue development and repair. Specifically, miR-10a-5p and hsa-miR-10b-5p are involved in vascular remodeling and angiogenesis by regulating endothelial cell migration and function [50]. miR-29a-3p contributes to extracellular matrix rearrangement by facilitating tissue reorganization and cell growth. During the remodeling phase, these miRNAs regulate fibroblast differentiation, collagen deposition, and wound contraction, directly affecting collagen expression [51,52]. Lastly, miR-21 promotes the migration of keratinocytes and fibroblasts, enhances cell survival, proliferation, and angiogenesis, while its inhibition delays re-epithelialization and wound contraction [53,54]. The cargo of fibroblast-derived EVs, enriched with key miRNAs, highlights their potential as bioactive agents capable of enhancing tissue repair and promoting optimal healing outcomes by supporting vascular development, cell survival, and tissue homeostasis.

### 3.4. Characterization of Bioactive Hydrogel

**Figure 5:**
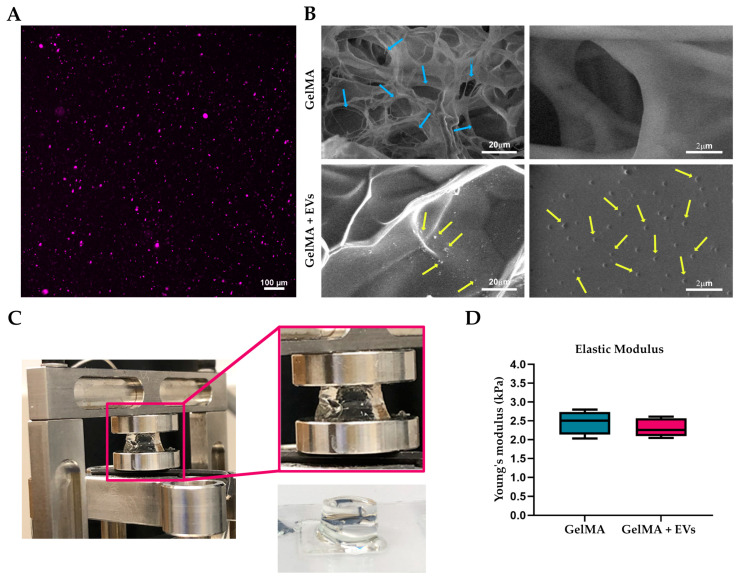
Bioactive hydrogel characterization. (**A**) Representative fluorescence images of bioactive bioink. EVs labeled with Cell Mask Deep Red (magenta) plasma membrane stain were effectively encapsulated into GelMA hydrogel. Scale bars represent 100 μm. (**B**) SEM images of 5% GelMA-based hydrogel with and without EVs. Blue and yellow arrows show the porosity of the hydrogel and the morphology of EVs, respectively. The scale bars represent 20 μm and 2 μm. (**C**) Actual image of the GelMA hydrogel during compression test in the DMA Q800 Dynamic Mechanical Analyzer and after UV polymerization. (**D**) Values of the compressive modulus of elasticity as calculated from stress–strain curves for 5% GelMA hydrogels both pure and loaded with EVs.

GelMA hydrogels were analyzed by confocal microscopy, SEM, and mechanical tests to assess vesicle incorporation and the structural characteristics of the biomaterial. All experiments were performed using hydrogels loaded with extracellular vesicles at a concentration of 9 × 10^9^ EVs/mL. The confocal microscopy analysis of the EVs labeled with Cell Mask Deep Red revealed their clear visibility and uniform distribution within the hydrogel matrix (Figure 5A). SEM images also confirm the presence of EVs in the functionalized matrices in contrast to the pure matrices (Figure 5B). This analysis also visualized a porous network in both types of hydrogels, an essential feature for tissue integration. Mechanical compression tests (Figure 5C) showed that the incorporation of EVs did not significantly affect the Young’s modulus value, with both pure GelMA hydrogels and those loaded with EVs presenting values between 2 and 2.5 kPa (Figure 5D). This stability highlights that the mechanical properties of GelMA remain unchanged despite EV incorporation, making these hydrogels very suitable for soft tissue engineering applications where mechanical integrity is critical. The stiffness of the skin, characterized by Young’s modulus in the range optimal for maintaining cell viability and promoting tissue regeneration [55], aligns well with the mechanical properties of GelMA hydrogels, further supporting their application in regenerating the target tissue.

### 3.5. Evaluation of EVs-Loaded Hydrogels in Skin Wound Healing In Vivo

**Figure 6:**
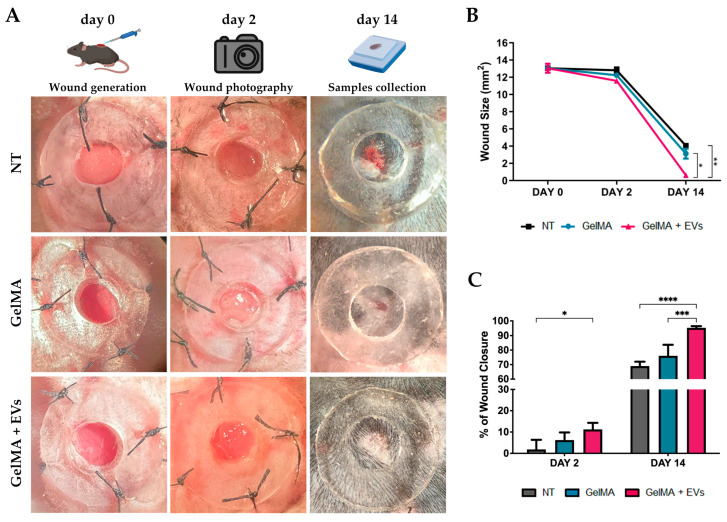
Wound healing in vivo. (**A**) Representative images displaying the wound healing process of mice under the three experimental conditions: NT, GelMA, and GelMA + EVs (day 0, 2, and 14). The rendering figures at the top, “wound generation”, “wound photography” and “sample collection” were created with BioRender.com. (**B**) Graph of wound closure progress in mice under the diverse experimental contexts: NT, GelMA, and GelMA + EVs from day 0 to day 14. Error bars represent ± SD. Two-Way ANOVA, * *p* < 0.05, ** *p* < 0.01. (**C**)Percentage of wound closure in mice in the different treatments: NT, GelMA, and GelMA + EVs. Error bars represent ± SD. Two-Way ANOVA, * *p* < 0.05, *** *p* < 0.001, **** *p* < 0.0001.

This study focused on evaluating the in vivo efficacy of fibroblast-derived EV-functionalized hydrogels. To this end, dorsal skin wounds were created on C57/BL6 mice and treated with (i) saline solution (NT), (ii) GelMA, or (iii) GelMA + EVs (9 × 10^9^ particles/mL). Wound healing was assessed via macroscopic analysis on days 2 and 14 post-treatment, supported by histological data (Figure 6A,B). On day 2, wounds treated with GelMA + EVs showed a more marked reduction in wound area compared to the other groups, with a mean wound area of 11.5 mm^2^ in the GelMA + EVs group versus 12.8 mm^2^ and 12.2 mm^2^ for the NT and GelMA groups, respectively. This trend persisted through day 14, where wounds in the GelMA + EVs group exhibited almost complete closure with a mean wound area of 0.62 mm^2^, which was significantly smaller compared to 4.4 mm^2^ in the NT group and 3.12 mm^2^ in the GelMA group. The quantitative analysis of wound area and closure rate revealed a statistically significant improvement in closure for GelMA + EVs compared to NT on day 2. By day 14, GelMA + EVs showed a significantly higher closure rate than both GelMA and NT (Figure 6C), underscoring the enhanced healing efficacy of EV-functionalized hydrogels across the healing timeline. These findings suggest that EVs support critical stages of the regenerative process, comprising inflammation resolution, angiogenesis, granulation tissue formation, and ECM remodeling, highlighting EV-functionalized hydrogels as a promising therapeutic approach for enhanced wound healing.

### 3.6. Regenerative Effects of Bioactive Hydrogels in Skin Wound Healing

**Figure 7:**
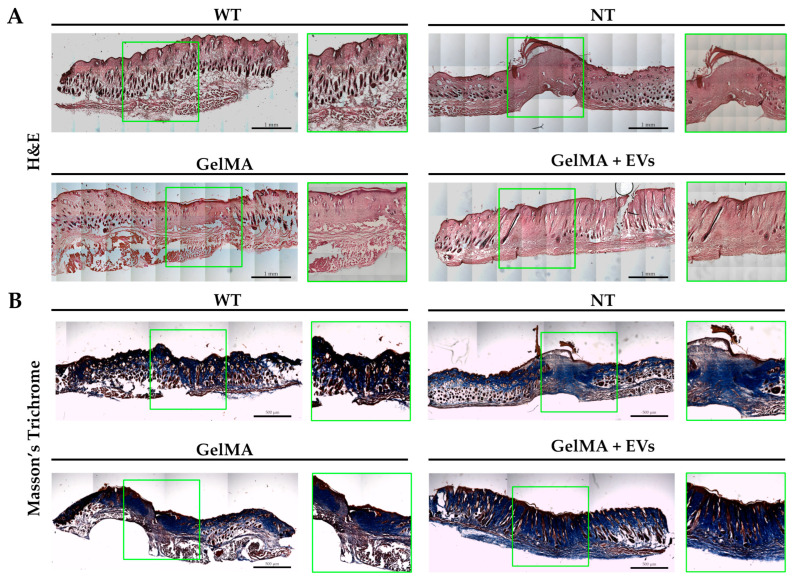
Histological analysis of skin. (**A**) H&E staining of wound sections WT, NT, GelMA, and GelMA + EVs groups at day 14. The scale bars represent 1 mm. (**B**) Masson’s trichrome staining of wound sections WT, NT, GelMA, and GelMA + EVs groups at day 14. The scale bars represent 500 μm.

**Figure 8:**
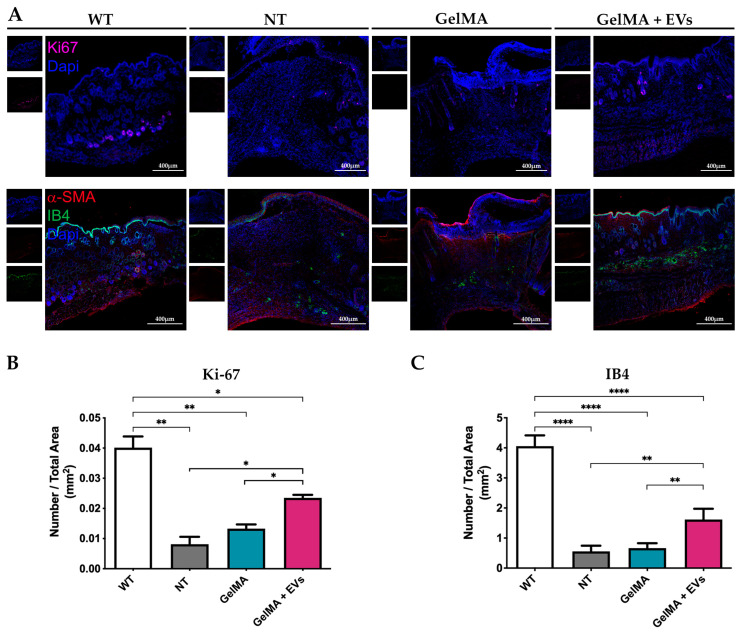
Immunofluorescence analysis on skin. (**A**) Representative immunofluorescence images of staining against Ki-67 (magenta) on skin sections of the four experimental conditions WT, NT, GelMA, and GelMA + EVs at day 14. The scale bars represent 400 μm. Nuclei were counterstained with DAPI (blue) (**upper panels**). Representative immunofluorescence images of staining against α-SMA (red) and IB4 (green) on skin sections of the different experimental conditions WT, NT, GelMA, and GelMA + EVs at day 14. The scale bars represent 400 μm. Nuclei were counterstained with DAPI (blue) (**lower panels**). (**B**,**C**) Diagrams represent the ratio between the number of Ki-67 positive cells (**B**) or the number of ISO/B4 positive capillaries (**C**) and the total area (mm2) in the different experimental groups. Error bars represent ± SD. Two-Way ANOVA, * *p* < 0.05, ** *p* < 0.01, **** *p* < 0.0001.

The hematoxylin–eosin (H&E) staining revealed distinct tissue organization among the NT, GelMA, and GelMA + EVs groups when compared to healthy tissue (WT). The GelMA + EVs group exhibited a denser cellular arrangement and more mature tissue architecture than the GelMA and NT groups on day 14 post-treatment. Specifically, the NT group displayed delayed and non-functional wound closure, characterized by extensive fibrotic areas at the injury site (Figure 7A). In contrast, structures resembling normal skin appendages (e.g., hair follicles, sebaceous glands, and sweat glands) were clearly visible in the GelMA + EVs group, whereas they were sparse or absent in the GelMA and NT groups. The epidermis was notably thin or absent in the NT group, while GelMA-treated wounds displayed partial epidermal regeneration without full re-epithelialization. Notably, the GelMA + EVs group achieved both full epidermal and dermal regeneration, closely mirroring the architecture of WT tissue. These findings suggest that the presence of EVs enhances cellular communication, promotes cell proliferation, and regulates ECM deposition, supporting an organized healing process more akin to natural tissue architecture [56,57]. Further validation by Masson’s trichrome evaluation demonstrated improved structural organization in the GelMA + EVs group, aligning with WT tissue composition. The analysis revealed minimal collagen disarray in GelMA + EVs-treated tissue, in contrast to the NT and GelMA groups, which exhibited prominent collagen deposits indicative of fibrotic scar formation (Figure 7B). Additionally, the GelMA + EVs-treated tissues retained the characteristic epidermal and dermal functions of WT tissue, underscoring its regenerative potential. These data suggest that our bioactive hydrogel offers a promising avenue for effective tissue regeneration by fostering re-epithelialization and controlled collagen deposition, essential for preserving skin integrity and function [58]. These observations were further supported by immunofluorescence staining for Ki-67, an established marker of proliferating cells. WT samples exhibited Ki-67 in the basal epidermis near hair follicles, a pattern replicated in the GelMA + EVs group (Figure 8A), indicating an enhanced cell proliferation relative to NT and GelMA groups (Figure 8B). This result reflects the ability of the bioactive hydrogel to support the cellular proliferation necessary for transitioning from granulation tissue to functional tissue remodeling [59]. Finally, immunofluorescence staining for α-SMA and isolectin B4, markers for myofibroblasts and endothelial cells, respectively, confirmed heightened vascularization in the GelMA + EVs group on day 14. The GelMA + EVs-treated tissue showed a marked increase in blood vessel density relative to NT and GelMA groups, which is critical for nutrient and oxygen supply during tissue repair (Figure 8C). The restored vascular network supports normal skin functions and underscores the bioactive hydrogel’s role in facilitating comprehensive skin regeneration, thus offering an effective strategy for improved wound healing outcomes [60]. It should be noted that some nonspecific signals observed in the immunofluorescence staining could be attributed to the composition of the epidermis, which includes desquamated and non-viable keratinocytes. Additionally, potential background autofluorescence or antibody cross-reactivity in these complex tissue sections may have contributed to the observed signals.

## 4. Conclusions

Wound healing remains a complex and challenging process, particularly in chronic or severe wounds, where the natural healing mechanisms are often impaired. Functionalized hydrogels incorporating EVs represent a promising solution to enhance tissue regeneration, offering a cell-free, immune-tolerant approach. Our results indicate that the hydrogels functionalized with fibroblast-derived EVs significantly accelerated skin repair in an immunocompetent mouse model, promoting the formation of skin appendages, improving tissue architecture, and reducing fibrosis. Importantly, this delivery strategy supports the sustained release of bioactive EVs at the wound site, addressing the limitations of previous EV-based therapies that struggled with poor retention [61,62,63]. Furthermore, the application of freeze-drying with lyoprotectants successfully preserved the structural integrity and bioactivity of EVs, overcoming key challenges related to long-term storage and transport and allowing immediate wound management This approach holds significant promise for the scalability and clinical translation of EV-based therapies, making them more accessible and practical for use in diverse medical environments. Specifically, it provides solutions for peculiar acute wounds, such as combat injuries, which require rapid and straightforward interventions. Moreover, one key finding from the miRNome analysis of EVs is the significant involvement of miRNAs in the wound healing process. In particular, several highly expressed miRNAs, including miR-10a-5p, miR-10b-5p, miR-29a-3p, and miR-21-5p, were identified. These results highlight the functional relevance of EV-derived miRNAs in orchestrating complex biological processes associated with tissue repair. Further investigation into their specific molecular mechanisms could provide valuable insights into the development of miRNA-based therapeutic strategies for enhanced wound healing and regenerative medicine. The generation of biofunctional hydrogel enriched with EVs represents a significant advancement in the field of regenerative medicine, combining the strengths of bioengineering, materials science, and cellular biology. This bioengineered scaffold not only enhances the speed and quality of healing but also supports the development of more functional tissue. Future work should focus on optimizing this system for human applications and exploring its utility in treating complex wounds or large-scale tissue damage. The potential to develop portable, EV-loaded hydrogels for immediate, on-site application could have profound implications for military medicine, emergency care, and other areas where rapid intervention is critical. This approach also holds potential for chronic wound management. Chronic wounds often arise as complications from conditions such as vascularization insufficiency, diabetes, and neuropathies. They are typically characterized by a prolonged inflammatory phase that hinders the natural healing process. The miRNome profile results support the potential use of our functionalized hydrogel to regulate persistent inflammation, thereby preventing complications such as infections or limb amputations.

Additionally, investigating the production of EVs from cells under stress conditions [24] could open new avenues for enhancing the therapeutic potential of this system. Stress-induced EVs may carry unique bioactive molecules that can further modulate the healing process, improve cellular communication, and promote tissue regeneration. Another promising direction could involve the development of multilayered hydrogels composed of layers with distinct biomechanical properties and functionalized with EVs derived from diverse cellular sources. This approach would allow the production of a tailored healing environment, enhancing the integration of the regenerating tissue while promoting functional recovery. By mimicking the complexity of native tissue architecture and combining the specialized bioactivity of EVs, such systems could redefine the landscape of advanced regenerative therapies. Our formulation is easy to produce and cost efficient since it does not require high-level biosafety laboratories. Moreover, it has the advantage of not being restricted to patient-specific EVs. This flexibility allows for the preparation of large quantities in advance, making it highly scalable for clinical applications. In conclusion, the study lays the foundation for a transformative approach to wound healing, seamlessly integrating advanced biological technologies with innovative biomaterials. By bridging, potentially, the gap between regenerative medicine and tissue engineering, our findings could open new horizons for developing innovative therapies that not only accelerate healing but also restore tissue functionality.

## Data Availability

The data supporting the findings of this article will be made available by the authors on request.
